# Comparison Between 3-Scan Trace and Diagonal Body Diffusion-Weighted Imaging Acquisitions: A Phantom and Volunteer Study

**DOI:** 10.18383/j.tom.2016.00229

**Published:** 2016-12

**Authors:** Stefanie J. Hectors, Mathilde Wagner, Idoia Corcuera-Solano, Martin Kang, Alto Stemmer, Michael A. Boss, Bachir Taouli

**Affiliations:** 1 Translational and Molecular Imaging Institute, Icahn School of Medicine at Mount Sinai, New York, New York;; 2 Department of Radiology, Icahn School of Medicine at Mount Sinai, New York, New York;; 3 Department of Medicine, Icahn School of Medicine at Mount Sinai, New York, New York;; 4 Siemens AG, Medical Solutions, Magnetic Resonance, Erlangen, Germany; and; 5 Applied Physics Division, National Institute of Standards and Technology, Boulder, Colorado

**Keywords:** diffusion-weighted imaging, phantom, volunteers, apparent diffusion coefficient

## Abstract

Diagonal diffusion-weighted imaging (dDWI) uses simultaneous maximized application of 3 orthogonal gradient systems as opposed to sequential acquisition in 3 directions in conventional 3-scan trace DWI (tDWI). Several theoretical advantages of dDWI vs. tDWI include reduced artifacts and increased sharpness. We compared apparent diffusion coefficient (ADC) quantification and image quality between monopolar dDWI and tDWI in a dedicated diffusion phantom (b = 0/500/900/2000 s/mm^2^) and in the abdomen (b = 50/400/800 s/mm^2^) and pelvis (b = 50/1000/1600 s/mm^2^) of 2 male volunteers at 1.5 T and 3.0 T. Phantom estimated signal-to-noise ratio (eSNR) was also measured. Two independent observers assessed the image quality on a 5-point scale. In the phantom, image quality was similar between tDWI and dDWI, with equivalent ADC quantification (mean coefficient of variation [CV] between sequences: 1.4% ± 1.2% at 1.5 T and 0.7% ± 0.7% at 3.0 T). Phantom eSNR was similar for both tDWI and dDWI, except for a significantly lower eSNR for b900 of dDWI at 3.0 T (*P* = .006). In the volunteers, the CV values between tDWI and dDWI were higher than those in the phantom (CV range: abdominal organs, 1.3%–13.3%; pelvic organs, 0.6%–5.7%). A trend toward significant better image quality for dDWI compared with tDWI was observed for b800 (abdomen) at 3.0 T and for b1000 and b1600 (pelvis) at 1.5 T (*P* = .063 to .066). Our data suggest that dDWI may provide better image quality than tDWI without affecting ADC quantification, needing confirmation in a future clinical study.

## Introduction

Diffusion-weighted imaging (DWI) is widely used for disease assessment, particularly in cancer. Cancerous tissues are often characterized by high cellularity, leading to hampered water diffusion, which can be quantitatively probed by measuring the apparent diffusion coefficient (ADC) (1). DWI is being increasingly used for the entire body rather than for only the brain, where it was applied initially (1–4). Major technical advancements, both hardware and software, have initiated the shift toward extracranial applications (5). DWI is most commonly performed in the clinic by single-shot spin-echo echo planar imaging (EPI) sequence with incorporated diffusion gradients (4). To account for diffusion anisotropy, usually a 3-scan trace approach (tDWI) is used, in which acquisitions are performed using 3 orthogonal diffusion gradient directions sequentially. Subsequently, orientation-invariant trace-weighted images are calculated by taking the geometric mean of the 3 signals. The tDWI approach has some disadvantages. First, the sequential application of gradients does not use full gradient strength, leading to relatively long echo times (TE) (6). In addition, there may be differences in eddy currents between the 3 acquisitions, leading to blurring in the trace-weighted images (7). An alternative for tDWI, called tetrahedral DWI, has been proposed, which uses full gradient strength, leading to shorter TE (8). However, tetrahedral DWI needs the acquisition of 4 diffusion directions compared with 3 in tDWI, and therefore, eddy currents induced by switching the diffusion gradients may also impair the trace-weighted images.

Another possible alternative for tDWI is diagonal DWI (dDWI), which, similar to tetrahedral DWI, uses simultaneous maximum diffusion gradient strength in all 3 directions for the highest b value. However, in contrast to tetrahedral DWI, in dDWI, diffusion is measured in only a single direction. The following are the 2 different types of dDWI techniques: one uses a specific bipolar isotropic gradient scheme that accounts for diffusion anisotropy (9), and the other uses simultaneous maximum application of monopolar diffusion gradients in all 3 orthogonal directions. The latter technique is available in clinical magnetic resonance imaging (MRI) systems (3-dimensional diagonal with Siemens Healthcare, 3-in-1 with GE Healthcare, and gradient overplus with Philips Healthcare). Because diffusion is measured in only a single direction (the net direction of all gradients), this dDWI technique is primarily applicable in organs in which diffusion is isotropic. The maximized gradient strength in the dDWI technique leads to relatively shorter TE (6) and potential elimination of blurring effects caused by differences in eddy currents (10). The current literature on dDWI is limited, with only a few reports on the use of monopolar dDWI in liver (11), soft tissue tumors (12), and spinal cord (6). There are no studies in which tDWI and dDWI are compared in terms of image quality and ADC quantitation.

The aim of this preliminary study is to compare tDWI and dDWI in a dedicated diffusion phantom and in the abdomen/pelvis of 2 healthy volunteers. We hypothesize that dDWI provides better image quality (secondary to reduction of eddy current artifacts) and higher signal-to-noise ratio (SNR) (given the lower TE) without affecting ADC quantification. We believe that this initial study is an important step toward validation of DWI in future multicenter clinical trials in cancer.

## Methodology

### Study Design

Monopolar tDWI and dDWI were quantitatively and qualitatively compared in a dedicated diffusion phantom and in the abdomen and pelvis of 2 volunteers. Quantitative comparison was performed by assessing ADC and SNR values in regions of interest (ROIs). In addition, test–retest measurements were performed in the phantom to assess repeatability of the ADC measurements using tDWI and dDWI. In this preliminary study, only single tDWI and dDWI measurements were performed in the volunteers, without test–retest repeatability assessment, to obtain initial measurements on ADC quantification and image quality using both techniques. Qualitative assessment was performed by 2 independent readers by scoring the images in terms of image sharpness, distortion, and artifacts.

### Phantom

Phantom MRI measurements were performed using a dedicated spherical diffusion phantom (High Precision Devices, Inc., Boulder, Colorado) consisting of 13 vials with varying mass fractions of polyvinylpyrrolidone (PVP). The distribution of PVP mass fractions in the phantom was as follows: 0% (vials 1–3), 10% (vials 4–5), 20% (vials 6–7), 30% (vials 8–9), 40% (vials 10–11), and 50% (vials 12–13). The vials are surrounded by ice water to maintain a temperature of 0°C during the MRI measurements to exclude thermal variation in ADC. Temperature of the phantom was measured before and after each scan.

### Volunteers

The volunteer part of the study was approved by our Institutional Review Board and is compliant with the Health Insurance Portability and Accountability Act. Informed consent was obtained before scanning. Two healthy male volunteers (ages, 22 and 23 years) were included.

### MRI Acquisition

MRI measurements were performed at 1.5 T (Aera, Siemens Healthineers, Erlangen, Germany) and 3.0 T (Skyra, Siemens Healthineers, Erlangen, Germany). Acquisition parameters of the phantom and volunteer scans are detailed in Table 1. All diffusion sequences consisted of a single-shot spin-echo EPI sequence with monopolar diffusion preparation (13). The number of averages of the dDWI sequence was multiplied by a factor of 3 compared with those of the tDWI sequence, except for b0 acquisitions, to account for the loss in SNR with a factor of √3 due to the single diffusion acquisition compared with the sequential acquisition of 3 diffusion directions in tDWI. The b values for the phantom were chosen as proposed by the phantom's manufacturer. For the volunteer study, b values recommended for abdomen and prostate examinations (14) were used. Acquisition parameters were matched as closely as possible between 1.5 T and 3.0 T. The shortest possible TE was chosen for both systems.

**Table 1.** Acquisition Parameters for tDWI and dDWI Pulse Sequences in Diffusion Phantom and In Vivo

	Phantom		In Vivo (Abdomen)		In Vivo (Pelvis)	
	tDWI	dDWI	tDWI	dDWI	tDWI	dDWI
Orientation	Coronal	Coronal	Axial	Axial	Axial oblique	Axial oblique
Diffusion scheme	Monopolar	Monopolar	Monopolar	Monopolar	Monopolar	Monopolar
Gradient directions [x,y,z]	[1.0, 1.0, −0.5], [1.0, −0.5, 1.0],[−0.5, 1.0, 1.0]	[1.0, 1.0, 1.0]	[1.0, 1.0, −0.5], [1.0, −0.5, 1.0],[−0.5, 1.0, 1.0]	[1.0 1.0 1.0]	[1.0, 1.0, −0.5], [1.0, −0.5, 1.0],[−0.5, 1.0, 1.0]	[1.0, 1.0, 1.0]
Read-out	SS SE-EPI	SS SE-EPI	SS SE-EPI	SS SE-EPI	SS SE-EPI	SS SE-EPI
b Values (s/mm^2^)	0, 500, 900, 2000	0, 500, 900, 2000	50, 400, 800	50, 400, 800	50, 1000, 1600	50, 1000, 1600
Averages	4, 4, 4, 4	4, 12, 12, 12	1, 2, 4	3, 6, 12	1, 5, 10	3, 15, 30
TE (ms)	110 (1.5 T); 99 (3.0 T)	106 (1.5 T); 95 (3.0 T)	73 (1.5 T); 70 (3.0 T)	70 (1.5 T); 68 (3.0 T)	83 (1.5 T); 71 (3.0 T)	79 (1.5 T); 69 (3.0 T)
TR (ms)	10 000	10 000	5000	5000	5000	5000
Slice thickness (mm)	5	5	5	5	5	5
Number of slices	5	5	30	30	20	20
Matrix	128 × 128	128 × 128	128 × 128	128 × 128	114 × 114	114 × 114
FOV (mm^2^)	210 × 210	210 × 210	370 × 250	370 × 250	250 × 250	250 × 250
GRAPPA acceleration	2	2	2	2	2	2
Fat suppression	None	None	SPAIR	SPAIR	SPAIR	SPAIR
Acquisition time (min)	7:10	7:10	2:12	2:12	4:27	4:27

The in vivo acquisitions were performed during free breathing.

Abbreviations: dDWI, diagonal diffusion-weighted imaging; FOV, field of view; GRAPPA, generalized autocalibrating partial parallel acquisition; SPAIR, spectral attenuated inversion recovery; tDWI, 3-scan trace diffusion-weighted imaging; SS SE-EPI, single-shot spin-echo echo planar imaging; TE, echo time; TR, repetition time.

For phantom acquisitions, a 20-channel head-and-neck coil was used. The phantom protocol started with a 3-plane localizer sequence to ensure exact positioning of the phantom in the isocenter in all 3 orthogonal directions. Subsequently, tDWI and dDWI were performed. The phantom measurements were performed in duplicate to assess test–retest repeatability. The phantom was repositioned between the test and retest measurements, and new scanner adjustments were made.

The 2 volunteers underwent scanning using a 32-channel body array coil. The subjects were asked to fast for 4 hours before the scanning, and to ensure a similar hydration level, subjects were asked to drink 1 L of water 1–2 hours before the scan. The abdominal and pelvic examinations included a 3-plane localizer sequence, followed by axial T2 HASTE (half-Fourier acquisition single-shot turbo spin-echo) sequence, tDWI, and dDWI.

### Quantitative Image Analysis

ADC maps were generated by fitting a linear model to the logarithmic signal data at different b values using a custom-written script in MATLAB R2015a (MathWorks Inc., Natick, Massachusetts). For the phantom measurements, square ROIs of 7 × 7 pixels were positioned using Matlab in each vial on the center section of the b0 image and subsequently propagated to other b values, avoiding the edges of the vials. The same ROIs were used for tDWI and dDWI. In the volunteers, 23 different ROIs were positioned using OsiriX (Pixmeo, Bernex, Switzerland) by observer 1 (MW), a radiologist with 4 years of experience in body MRI in the following organs: liver (segments III, IV, VI, and VIII and central right liver), gallbladder, pancreas (head, body, and tail), spleen (mid-anterior and mid-posterior poles), kidneys (right and left anterior and posterior cortex and anterior and posterior medulla), prostate (right and left peripheral zone and transitional zone), and urinary bladder. Similar to the phantom data, the ROIs were positioned on the DWI images with the lowest b value (b50), with reference to the T2 HASTE images, and subsequently propagated to other b values. Adjustments to the ROIs in the volunteers were done if necessary, for example, because of either motion or distortion between the acquisitions. The same ROI adjustments were done for tDWI and dDWI, such that the same ROI was used for analysis of both methods. Average ADC and ranges of ADC values within the ROI (maximum minus minimal ADC value) were recorded for each ROI. In addition, for the phantom DWI data estimated SNR (eSNR), values were recorded for each ROI by dividing the average signal in the ROI by the standard deviation of the signal as previously proposed for SNR measurement in the presence of parallel imaging (15, 16).

### Qualitative Image Analysis

Observer 1 along with observer 2 (ICS), a radiologist with 2 years of experience in body MRI, assessed the image quality of all phantom and volunteer diffusion acquisitions. The diffusion-weighted images were scored for anatomical distortion, image sharpness, and artifacts using a 5-point Likert scale (1, unacceptable; 2, poor; 3, satisfactory; 4, good; and 5, excellent). In addition, the overall quality of the ADC map was scored on the same scale.

### Statistical Analysis

All data are presented as mean ± standard deviation. Variability and differences in phantom ADC values between tDWI and dDWI, between 1.5 T and 3.0 T, and between test–retest measurements were assessed using the coefficient of variation (CV). Additional assessment of test–retest repeatability was performed using Bland–Altman analysis, consisting of assessment of the coefficient of repeatability (CR) and Bland–Altman limits of agreement (BA-LA) between the tDWI and dDWI ADC values of the test–retest measurements. Wilcoxon signed rank tests were used to compare eSNR values between tDWI and dDWI at b900 and b2000 in the phantom at 1.5 T and 3.0 T. ADC ranges of tDWI and dDWI were also compared using Wilcoxon signed rank tests. For the volunteer data, the CV of ADC values between tDWI and dDWI was determined for each ROI. In addition, the CV values between ADC values at 1.5 T and 3.0 T were determined. Differences in the image quality scores between tDWI and dDWI in the volunteer data were compared using Wilcoxon signed rank tests. For all statistical tests, a *P* value of <.05 was considered significant. All the statistical analyses were performed in Matlab.

## Results

### Phantom Data

#### Quantitative Results.

For test–retest measurements, the average ADC values in each vial in each system are displayed in Table 2. In addition, the CV values between test and retest measurements, between tDWI and dDWI and between field strengths are shown. ADC values were similar between tDWI and dDWI (mean CV: 1.4% ± 1.2% at 1.5 T; 0.7% ± 0.7% at 3 T), between test and retest measurements (mean CV: <2.2% at both field strengths for tDWI and dDWI), and between field strengths (mean CV: 1.5% ± 2.2% for tDWI; 1.4% ± 1.1% for dDWI).

**Table 2.** Average ADC and CV Values for Phantom Test-Retest Acquisitions

Vial No.	PVP %	1.5 T							3.0 T							1.5 T vs. 3.0 T	
		tDWI			dDWI			CV tDWI–dDWI	tDWI			dDWI			CV tDWI–dDWI	tDWI	dDWI
		ADC Test	ADC Retest	CV Test–Retest	ADC Test	ADC Retest	CV Test–Retest		ADC Test	ADC Retest	CV Test–Retest	ADC Test	ADC Retest	CV Test–Retest		CV 1.5 T–3.0 T	CV 1.5 T–3.0 T
1	0	1.13 ± 0.01	1.13 ± 0.02	0.0	1.14 ± 0.02	1.15 ± 0.02	1.0	0.1	1.13 ± 0.01	1.13 ± 0.01	0.1	1.13 ± 0.01	1.14 ± 0.01	0.7	0.2	0.0	0.5
2	0	1.12 ± 0.02	1.13 ± 0.02	0.6	1.12 ± 0.02	1.14 ± 0.02	1.3	0.2	1.13 ± 0.01	1.12 ± 0.01	0.4	1.12 ± 0.01	1.15 ± 0.01	1.6	0.7	0.3	0.0
3	0	1.15 ± 0.01	1.17 ± 0.02	1.5	1.13 ± 0.01	1.16 ± 0.02	1.4	1.0	1.14 ± 0.01	1.16 ± 0.03	1.4	1.13 ± 0.01	1.17 ± 0.01	2.4	0.5	0.6	0.5
4	10	0.86 ± 0.01	0.88 ± 0.01	1.8	0.86 ± 0.01	0.88 ± 0.01	1.6	0.1	0.86 ± 0.01	0.87 ± 0.01	0.6	0.85 ± 0.01	0.87 ± 0.01	2.0	1.0	1.2	1.6
5	10	0.85 ± 0.01	0.88 ± 0.01	2.4	0.85 ± 0.01	0.88 ± 0.01	2.5	0.3	0.84 ± 0.01	0.87 ± 0.01	2.2	0.84 ± 0.01	0.88 ± 0.01	3.0	0.1	0.7	0.5
6	20	0.63 ± 0.01	0.64 ± 0.01	1.0	0.64 ± 0.01	0.65 ± 0.01	1.5	0.9	0.63 ± 0.01	0.64 ± 0.00	0.9	0.63 ± 0.01	0.64 ± 0.01	1.8	0.4	1.3	1.8
7	20	0.63 ± 0.00	0.65 ± 0.01	1.7	0.66 ± 0.01	0.67 ± 0.01	1.4	2.3	0.62 ± 0.01	0.65 ± 0.01	2.7	0.64 ± 0.01	0.68 ± 0.01	3.6	2.5	1.2	0.5
8	30	0.42 ± 0.01	0.44 ± 0.01	2.5	0.44 ± 0.01	0.45 ± 0.01	2.7	2.1	0.43 ± 0.01	0.44 ± 0.01	1.7	0.43 ± 0.01	0.45 ± 0.01	2.1	0.5	0.2	1.2
9	30	0.41 ± 0.01	0.43 ± 0.01	2.5	0.43 ± 0.01	0.44 ± 0.01	2.1	2.3	0.41 ± 0.01	0.42 ± 0.01	2.4	0.41 ± 0.01	0.43 ± 0.01	2.8	0.4	0.7	2.2
10	40	0.25 ± 0.01	0.25 ± 0.01	1.0	0.26 ± 0.01	0.26 ± 0.01	0.0	1.7	0.24 ± 0.01	0.24 ± 0.01	1.4	0.24 ± 0.01	0.25 ± 0.01	3.9	0.1	2.8	2.8
11	40	0.23 ± 0.01	0.24 ± 0.01	2.0	0.24 ± 0.01	0.25 ± 0.01	1.8	1.5	0.23 ± 0.01	0.24 ± 0.01	1.5	0.23 ± 0.01	0.24 ± 0.01	1.8	0.1	0.9	2.2
12	50	0.13 ± 0.02	0.12 ± 0.02	1.1	0.12 ± 0.02	0.13 ± 0.02	2.1	2.1	0.13 ± 0.02	0.12 ± 0.02	4.0	0.12 ± 0.02	0.12 ± 0.02	1.6	1.0	1.4	0.7
13	50	0.13 ± 0.01	0.13 ± 0.02	1.6	0.12 ± 0.01	0.13 ± 0.01	4.8	4.2	0.12 ± 0.01	0.11 ± 0.01	1.0	0.12 ± 0.01	0.12 ± 0.01	1.2	1.1	8.7	3.9

ADC is expressed in 10^−3^ mm^2^/s. CV values (%) mentioned in the table are between test–retest acquisitions, between tDWI and dDWI, and between 1.5 T and 3.0 T.

Abbreviations: ADC, apparent diffusion coefficient; dDWI, diagonal diffusion-weighted imaging; PVP, polyvinylpyrrolidone; tDWI, 3-scan trace diffusion-weighted imaging.

**Figure 1.**
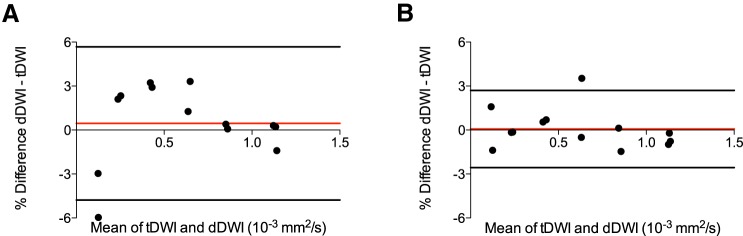
Bland–Altman plots comparing 3-scan trace diffusion-weighted imaging (tDWI) and diagonal diffusion-weighted imaging (dDWI) phantom apparent diffusion coefficient (ADC) measurements at 1.5 T (A) and at 3.0 T (B), illustrating similar ADC quantification between both techniques at both field strengths.

Bland–Altman plots of differences between tDWI and dDWI ADC measurements are shown in Figure 1 for both 1.5 T and 3.0 T, further illustrating high similarity in ADC quantification between both techniques (1.5 T: CR, 5.2% and BA-LA, −4.8% to 5.7%; 3.0 T: CR, 2.6%, BA-LA, −2.6% to 2.7%). The 2 vials with 50% PVP (and thus low ADC) exhibited the largest differences between tDWI and dDWI, particularly at 1.5 T (−3.0% and −6.0%, respectively).

Average eSNR values in the phantom vials in the b900 and b2000 images of tDWI and dDWI at 1.5 T and 3.0 T are displayed in Table 3. The eSNR values were similar for tDWI and dDWI, except for a significantly lower eSNR for dDWI for b900 at 3.0T (*P* = .006).

**Table 3.** Average eSNR Values in the Diffusion Phantom

Vial No.	1.5 T				3.0 T			
	eSNR_tDWI_ b900	eSNR_dDWI_ b900	eSNR_tDWI_ b2000	eSNR_dDWI_ b2000	eSNR_tDWI_ b900	eSNR_dDWI_ b900	eSNR_tDWI_ b2000	eSNR_dDWI_ b2000
1	55.9 ± 2.5	46.6 ± 0.9	31.7 ± 5.8	30.0 ± 1.7	49.9 ± 10.8	45.4 ± 9.8	43.3 ± 9.7	35.7 ± 6.3
2	32.9 ± 6.7	22.7 ± 1.4	24.2 ± 4.6	19.6 ± 2.1	28.3 ± 0.8	30.0 ± 7.3	27.1 ± 0.8	35.3 ± 7.3
3	23.7 ± 2.2	21.7 ± 0.6	20.3 ± 4.4	19.0 ± 1.2	23.3 ± 8.2	21.4 ± 8.5	18.9 ± 10.2	20.1 ± 8.4
4	47.4 ± 1.1	40.0 ± 8.9	37.0 ± 2.7	38.7 ± 7.3	64.8 ± 6.3	57.0 ± 5.3	68.4 ± 10.3	61.5 ± 12.5
5	48.5 ± 6.4	41.3 ± 4.8	45.1 ± 1.6	38.6 ± 5.3	20.0 ± 0.8	20.6 ± 3.5	20.6 ± 1.2	23.0 ± 6.4
6	67.2 ± 8.6	76.6 ± 22.2	58.4 ± 8.9	54.1 ± 14.8	82.5 ± 21.3	81.4 ± 9.0	98.6 ± 10.7	91.9 ± 16.5
7	18.1 ± 0.3	18.6 ± 0.7	18.6 ± 0.1	19.1 ± 1.4	18.5 ± 1.8	17.7 ± 1.1	18.3 ± 1.8	17.1 ± 0.2
8	41.0 ± 4.1	42.0 ± 1.6	30.2 ± 2.8	42.2 ± 2.8	40.5 ± 4.1	40.0 ± 4.9	41.2 ± 2.8	41.5 ± 3.3
9	27.3 ± 3.0	28.3 ± 4.2	29.8 ± 1.3	29.1 ± 2.8	35.9 ± 3.0	30.0 ± 2.7	38.4 ± 6.1	32.7 ± 3.3
10	30.2 ± 2.1	32.7 ± 3.0	30.5 ± 3.4	32.7 ± 0.4	40.5 ± 7.1	32.3 ± 0.3	38.0 ± 2.6	31.0 ± 0.7
11	28.9 ± 1.1	32.7 ± 4.0	30.9 ± 1.6	34.4 ± 0.8	48.0 ± 0.1	42.8 ± 6.4	51.4 ± 0.8	44.5 ± 8.8
12	46.5 ± 0.8	37.1 ± 6.2	48.2 ± 7.4	40.5 ± 2.6	40.2 ± 1.9	39.6 ± 3.3	69.3 ± 2.7	49.2 ± 4.6
13	42.5 ± 2.3	40.9 ± 4.9	48.0 ± 1.5	46.5 ± 3.1	65.7 ± 3.6	60.5 ± 1.5	54.0 ± 4.1	64.9 ± 21.4
Mean	39.2 ± 13.9	37.0 ± 14.8	35.6 ± 11.9	34.2 ± 10.8	42.7 ± 19.2	39.9 ± 18.1	45.2 ± 23.4	42.2 ± 20.8

Average SNR values are listed for tDWI and dDWI for the b900 and b2000 images in vitro at 1.5 T and 3.0 T. Wilcoxon signed rank *P* values between eSNR of tDWI and dDWI are 0.273, 0.340, 0.006, and 0.080 for b900 at 1.5 T, b2000 at 1.5 T, b900 at 3.0 T, and b2000 at 3.0 T, respectively.

**Figure 2.**
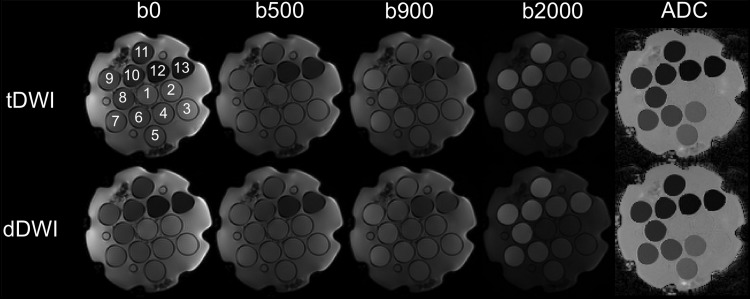
Representative diffusion-weighted imaging (DWI) images and ADC maps using tDWI and dDWI of a dedicated NIST diffusion phantom (containing 13 vials) at 1.5 T (see Table 1 for sequence parameters). Similar ADC quantification and image quality was observed between tDWI and dDWI.

Representative diffusion-weighted images and ADC maps of the phantom are shown in Figure 2, showing equal appearance for tDWI and dDWI. ADC variation within ROIs in the vials on these maps was determined by assessing the ADC value range. A trend was observed toward slightly broader ADC ranges for dDWI compared with those for tDWI in the phantom both at 1.5 T and 3.0 T (ADC range at 1.5 T: tDWI, 0.049 ± 0.019 × 10^−3^ mm^2^/s; dDWI, 0.051 ± 0.020 × 10^−3^ mm^2^/s, *P* = .057; ADC range at 3.0T: tDWI, 0.042 ± 0.022 × 10^−3^ mm^2^/s; dDWI, 0.048 ± 0.014 × 10^−3^ mm^2^/s, *P* = .057).

#### Qualitative Results.

Scores were overall higher at 1.5 T than at 3.0 T; however, there was no apparent difference between quality scores of tDWI and dDWI (Table 4).

**Table 4.** Average Quality Scores in the Diffusion Phantom

	1.5 T		3.0 T	
	tDWI	dDWI	tDWI	dDWI
b500				
Anatomic distortion	4.0 ± 0.0	4.0 ± 0.0	3.0 ± 0.0	3.0 ± 0.0
Image sharpness	4.0 ± 0.0	4.0 ± 0.0	3.5 ± 0.7	3.0 ± 0.0
Artifacts	4.0 ± 0.0	4.0 ± 0.0	4.0 ± 0.0	4.0 ± 0.0
Sum	12.0 ± 0.0	12.0 ± 0.0	10.5 ± 0.7	10.0 ± 0.0
b900				
Anatomic distortion	4.0 ± 0.0	4.0 ± 0.0	3.0 ± 0.0	4.0 ± 0.0
Image sharpness	4.5 ± 0.7	4.0 ± 0.0	3.5 ± 0.7	4.0 ± 0.0
Artifacts	4.0 ± 0.0	4.0 ± 0.0	4.0 ± 0.0	4.0 ± 0.0
Sum	12.5 ± 0.7	12.0 ± 0.0	10.5 ± 0.7	11.0 ± 0.0
b2000				
Anatomic distortion	4.5 ± 0.7	4.5 ± 0.7	4.0 ± 0.0	3.5 ± 0.7
Image sharpness	5.0 ± 0.0	5.0 ± 0.0	4.0 ± 0.0	4.5 ± 0.7
Artifacts	4.0 ± 0.0	4.0 ± 0.0	4.0 ± 0.0	4.0 ± 0.0
Sum	13.5 ± 0.7	13.5 ± 0.7	12.0 ± 0.0	12.0 ± 1.4
ADC				
Overall quality	4.5 ± 0.7	4.5 ± 0.7	3.0 ± 0.0	3.5 ± 0.7

The average quality scores are those from 2 observers for in vitro tDWI and dDWI measurements at 1.5 T and 3.0 T.

Abbreviations: ADC, apparent diffusion coefficient; dDWI, diagonal diffusion-weighted imaging; tDWI, 3-scan trace diffusion-weighted imaging.

### In Vivo Data

#### Quantitative Results.

**Figure 3.**
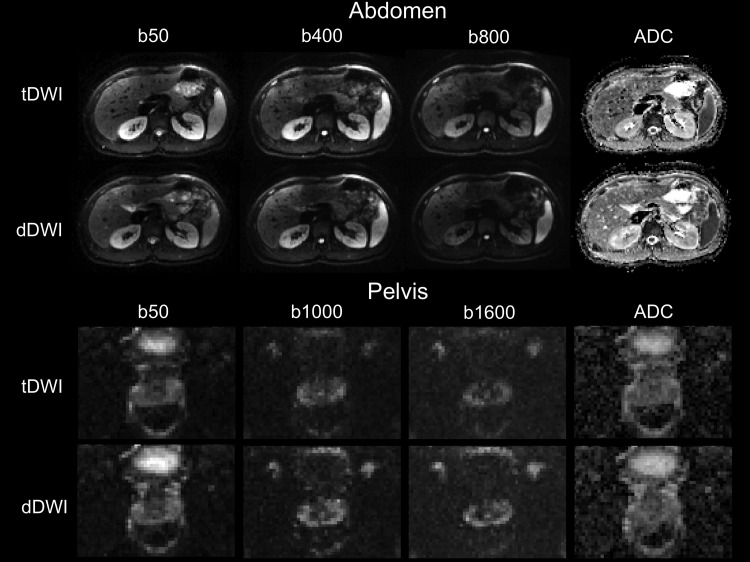
Representative images using tDWI and dDWI in the abdomen and pelvis of a 23-year-old male volunteer at 1.5 T. In the abdomen, the image quality was scored similar for both acquisitions (tDWI: image quality total score = 12.5 at b400, 12.5 at b800, and overall quality score = 5 for ADC; dDWI image quality total score = 12 at b400, 12 at b800, and overall quality score = 5 for ADC). In the pelvis, the image quality scored better in terms of image sharpness and artifacts for dDWI compared with tDWI (tDWI: image quality total score = 8 at b1000, 7 at b1600, and overall quality score = 3 for ADC; dDWI image quality total score = 10 at b1000, 10 at b1600, and overall quality score = 3.5 for ADC).

Representative diffusion-weighted images and ADC maps of tDWI and dDWI in the abdomen and pelvis of a healthy volunteer scanned at 1.5 T are displayed in Figure 3. Numbers of pixels and average ADC values in the ROIs drawn in both volunteers and the CV between ADC values of tDWI and dDWI and between 1.5 T and 3.0 T in each ROI are given in Table 5 for both systems. The CV values between tDWI and dDWI were lower for pelvic organs and in the liver, gallbladder, and kidneys (<7%), but higher values were found in the pancreas (up to 13%) and the spleen (up to 10%). The CV values between field strengths were in the same order of magnitude as the CV values between techniques (mean CV: 1.5 T vs. 3.0 T, <13.5%). Ranges of ADC values in the ROIs were not significantly different between tDWI and dDWI at both field strengths (1.5 T: range tDWI, 0.59 ± 0.31 × 10^−3^ mm^2^/s; dDWI, 0.55 ± 0.23 × 10^−3^ mm^2^/s, *P* = .429; 3.0 T: range tDWI, 0.54 ± 0.40 × 10^−3^ mm^2^/s; dDWI, 0.57 ± 0.32 × 10^−3^ mm^2^/s, *P* = .287).

**Table 5.** ROI Size Range, Average ADC and CV Values in the Abdomen and Pelvis

Tissue	ROI Size (Range of Number of Pixels)	1.5 T			3.0 T			CV (1.5 T vs. 3.0 T)	
		tDWI	dDWI	CV	tDWI	dDWI	CV	tDWI	dDWI
Liver	43–55	1.24 ± 0.08	1.28 ± 0.03	2.6	1.32 ± 0.06	1.20 ± 0.19	6.8	5.6	9.1
Gallbladder	20–28	3.02 ± 0.09	3.06 ± 0.33	4.0	3.22 ± 0.49	3.23 ± 0.09	6.3	9.1	6.9
Pancreas	16–39	1.39 ± 0.01	1.43 ± 0.09	2.6	1.63 ± 0.26	1.36 ± 0.25	13.3	10.9	12.4
Spleen	36–51	0.86 ± 0.04	0.77 ± 0.03	7.6	0.76 ± 0.15	0.73 ± 0.01	10.0	9.1	4.1
Renal cortex	2–5	1.94 ± 0.05	1.96 ± 0.10	1.3	1.91 ± 0.10	1.88 ± 0.04	1.7	1.4	2.9
Renal medulla	4–7	1.85 ± 0.04	1.93 ± 0.17	5.5	1.90 ± 0.07	1.98 ± 0.11	2.9	1.7	7.1
Prostate PZ	9–12	1.07 ± 0.05	1.11 ± 0.00	2.5	1.15 ± 0.05	1.11 ± 0.03	2.6	4.7	1.4
Prostate TZ	10–13	0.90 ± 0.04	0.86 ± 0.02	2.7	0.94 ± 0.01	0.99 ± 0.11	5.7	3.4	9.5
Urinary bladder	34–86	1.93 ± 0.04	1.94 ± 0.01	0.6	2.34 ± 0.09	2.21 ± 0.29	4.6	13.5	8.6

This table presents the ROI size range, average ADC values (10^−3^ mm^2^/s), and CV values (%) between tDWI and dDWI and between 1.5 T and 3.0 T in abdominal and pelvic organs of 2 healthy volunteers. For organs/tissues in which multiple ROIs were drawn (ie, liver, pancreas, spleen, renal cortex, renal medulla, and prostate peripheral zone), the average values of all ROIs are listed.

Abbreviations: dDWI, diagonal diffusion-weighted imaging; PZ, peripheral zone; ROI, region of interest; tDWI, 3-scan trace diffusion-weighted imaging; TZ, transitional zone.

#### Qualitative Results.

There was a trend toward an overall higher quality score for dDWI compared with tDWI for b800 of the abdomen scan at 3.0 T and for b1000 and b1600 of the pelvic scan at 1.5 T (*P* value range, from .063 to .066) (Table 6).

**Table 6.** Average Quality Scores in the Abdomen and Pelvis

	1.5 T			3.0 T		
	tDWI	dDWI	*P*^a^	tDWI	dDWI	*P*^a^
b400 abdomen						
Anatomic distortion	4.5 ± 0.0	4.3 ± 0.4	0.317	3.0 ± 0.0	3.3 ± 1.1	0.563
Image sharpness	4.0 ± 0.0	4.0 ± 0.0	1.000	3.0 ± 0.0	3.5 ± 0.0	0.157
Artifacts	4.0 ± 0.0	4.0 ± 0.0	1.000	3.0 ± 0.0	2.8 ± 0.4	0.317
Sum	12.5 ± 0.0	12.3 ± 0.4	0.317	9.0 ± 0.0	9.5 ± 0.7	0.414
b800 abdomen						
Anatomic distortion	4.5 ± 0.0	4.3 ± 0.4	0.317	2.8 ± 0.4	4.0 ± 0.0	0.059
Image sharpness	4.0 ± 0.0	4.0 ± 0.0	1.000	2.8 ± 1.1	3.8 ± 0.4	0.102
Artifacts	4.0 ± 0.0	4.0 ± 0.0	1.000	2.8 ± 0.4	3.3 ± 0.4	0.157
Sum	12.5 ± 0.0	12.3 ± 0.4	0.317	8.3 ± 1.8	11.0 ± 0.7	0.065
ADC abdomen						
Overall quality	5.0 ± 0.0	5.0 ± 0.0	1.000	3.0 ± 0.0	3.3 ± 0.4	0.317
b1000 pelvis						
Anatomic distortion	2.8 ± 0.4	3.0 ± 0.0	0.317	3.5 ± 0.7	3.0 ± 1.4	0.157
Image sharpness	2.0 ± 0.0	2.8 ± 0.3	0.083	3.5 ± 0.0	3.0 ± 1.4	0.414
Artifacts	2.5 ± 0.7	3.5 ± 0.7	0.102	3.5 ± 0.7	3.8 ± 0.4	0.317
Sum	7.3 ± 1.1	9.3 ± 1.1	0.063	10.5 ± 1.4	9.8 ± 3.2	0.276
b1600 pelvis						
Anatomic distortion	2.3 ± 0.4	3.0 ± 0.0	0.083	2.5 ± 0.7	2.5 ± 0.7	1.000
Image sharpness	2.0 ± 0.0	2.8 ± 0.4	0.083	2.5 ± 0.7	2.5 ± 0.7	1.000
Artifacts	2.3 ± 0.4	3.8 ± 0.4	0.063	2.8 ± 0.4	2.5 ± 0.7	0.563
Sum	6.5 ± 0.7	9.5 ± 0.7	0.066	7.8 ± 0.4	7.5 ± 2.1	0.705
ADC pelvis						
Overall quality	3.0 ± 0.0	3.5 ± 0.7	0.157	3.5 ± 0.7	3.8 ± 1.1	0.317

The average quality scores were calculated in the abdomen and pelvis using tDWI and dDWI measurements of 2 volunteers at 1.5 T and 3.0 T.

^a^Wilcoxon signed rank test.

## Discussion

Our preliminary study describes a qualitative and quantitative comparison between tDWI and dDWI, in a dedicated diffusion phantom and in the abdomen and pelvis in volunteers. The phantom data showed highly similar results for tDWI and dDWI. ADC quantification was the same for both techniques, which was anticipated, as the diffusion is expected to be fully isotropic in the phantom vials. Marginally higher differences between tDWI and dDWI ADC values were observed for the 2 vials with the lowest ADC values, primarily at 1.5 T. A reason for the higher variability for these vials could be that the diffusion decay curve is sampled only partially in these vials with the used b values, leading to ADC measurement inaccuracy. No increase in eSNR was found for dDWI compared with tDWI in the phantom. Although no substantial gain in SNR was anticipated because the difference in TE between tDWI and dDWI was only small in our systems, the significantly lower SNR for dDWI at b900 at 3.0 T was unexpected. A possible explanation could be that, in our study, only eSNR was measured because of the applied parallel imaging, which may have affected the accuracy of the SNR measurement. The most likely reason for the limited difference in TE between dDWI and tDWI was the use of orthogonal oblique diffusion directions in tDWI on the Siemens systems, with the use of higher gradient strength compared with the more traditional sequential use of the 3 gradient channels.

ADC variation, as expressed by the ADC range within the ROIs, was similar between dDWI and tDWI, with a marginal trend toward broader ADC range for dDWI at both systems, whereas, it was anticipated that heterogeneity of dDWI would be possibly lower, given the lower TE (and higher SNR) and reduced effects of eddy currents. However, the effects of eddy currents were most probably not substantial in the phantom measurements, given the high image quality scores for both tDWI and dDWI by the 2 observers.

The volunteer data showed higher differences in ADC values between tDWI and dDWI, particularly in the pancreas and spleen. These differences could be related to the test–retest variability within the same imaging session. The higher variability in vivo compared with the phantom data could be because of several factors such as respiratory motion (leading to variability in anatomical location of the ROI between both sequences) and cardiac pulsation artifacts, which could induce errors in diffusion parameter estimation (17). The cardiac pulsation artifacts may be partially corrected by the use of flow-compensated bipolar diffusion pulses (18). Braithwaite et al. studied the variability across 5 identical tDWI sequences in the abdomen in the same imaging session and reported a CV of ∼15% in the liver, pancreas, and spleen (19), which is in the same order of magnitude as the CV values found between tDWI and dDWI in our study. Nevertheless, the variability in the ADC values between tDWI and dDWI in the abdominal organs of the volunteers may also be partly caused by diffusion anisotropy in the abdominal organs, which is compensated for in tDWI but not in dDWI. Reports on diffusion anisotropy, particularly the liver, have been conflicting. Taouli et al. showed that diffusion is isotropic in the liver parenchyma (20), whereas Tosun et al. observed a significant nonzero value for fractional anisotropy (FA) in the liver (average FA, 0.48) using diffusion tensor imaging (DTI) measurements, indicative of substantial anisotropy in the liver parenchyma (21). Kidney diffusion anisotropy has been studied quite extensively with DTI, showing significant anisotropy, particularly in the renal medulla (FA range: 0.24–0.57 in the medulla compared with 0.13–0.31 in the cortex) (22). Diffusion in the pancreas has also been shown to be anisotropic (mean FA, 0.38) (23). There are no reports on DTI FA values in the spleen.

In general, dDWI using monopolar gradients should preferably not be applied in organs with anisotropic diffusion, as it may jeopardize accurate ADC quantification in these tissues. For such organs, dDWI using a bipolar isotropic diffusion scheme could possibly be used as an alternative (9). In the prostate, low CV values were observed, indicative of the absence of the effects of anisotropy, motion, and pulsatile flow, unlike the kidney and spleen. Indeed, FA is shown to be lower in the prostate than in the liver (average FA values of ∼0.17 in the peripheral zone and 0.24 in the transitional zone) (24, 25), indicative of relatively low diffusion anisotropy, and therefore, dDWI is likely more applicable in the prostate than in the abdominal organs. According to our initial results, we observed better image quality at b1000 and b1600 in the prostate at 1.5 T using dDWI; however, better image quality was not observed at 3.0 T. The latter finding could be explained by the fact that 1.5 T DWI MRI examinations in the pelvis generally suffer from low SNR, and therefore, the lower TE in dDWI than in tDWI likely has a more substantial effect on the image quality at 1.5 T compared with that at 3.0 T.

High reproducibility between ADC values at 1.5 T and 3.0 T was observed, both in the phantom and the volunteers. Earlier studies also have shown high similarity between ADC values at both field strengths in a dedicated diffusion phantom (26) and in the abdominal organs (27). In our study, keeping acquisition parameters identical between 1.5 T and 3.0 T, except for differences in TE, was intentional. For clinical application of the diffusion methods, the protocols could be further optimized specific for field strength, particularly taking into account the effect of the strength of the magnetic field on tissue T1 (28).

Our preliminary results comparing tDWI and dDWI in the abdominal and pelvic organs justify future studies applying dDWI in the body, particularly in the prostate, as our data suggest that dDWI may indeed allow for better image quality while maintaining equivalent ADC quantification. In addition, it would be interesting to compare the repeatability of the monopolar dDWI diffusion scheme with the bipolar isotropic dDWI (9) in organs that are sensitive to cardiac pulsatile motion and/or that exhibit diffusion anisotropy, such as the kidneys. In general, for future applicability of dDWI in clinical practice, the dDWI protocol should preferably be standardized across vendors, and exact reporting of the gradient scheme (eg, monopolar or isotropic bipolar) is needed for accurate assessment of the results.

Our study has some limitations. First, the sample size of the volunteer study was very small in this preliminary study. In addition, more accurate assessment of SNR could have been obtained when the phantom and volunteer acquisitions were performed without parallel imaging. Nevertheless, we intentionally applied parallel imaging because it is widely used clinically for DWI. An alternative accurate assessment of SNR could have been obtained if the test–retest measurements were performed within the same examination without repositioning. SNR measurement could than have been obtained by a 2-image SNR method (29). However, in our study, we intentionally repositioned the phantom between the measurements to better reflect test–retest repeatability in clinical practice, in which the subject would be scanned at different instances.

In conclusion, we have shown that both tDWI and dDWI provide similar ADC quantification and image quality in a dedicated diffusion phantom. In vivo, preliminary data suggested a trend toward better image quality for dDWI, particularly in the pelvis, which should be confirmed in a larger study with test–retest measurements.
